# Evolution of Communities in the Medical Sciences: Evidence from the Medical Words Network

**DOI:** 10.1371/journal.pone.0167546

**Published:** 2016-12-02

**Authors:** Amir H. Shirazi, Arash Badie Modiri, Sara Heydari, Jennifer L. Rohn, Gholam R. Jafari, Ali R. Mani

**Affiliations:** 1 Department of Physics, Shahid Beheshti University - G.C., Tehran, Iran; 2 Centre for Nephrology, UCL Division of Medicine, Royal Free Campus, University College London, London, United Kingdom; 3 Institute for Liver and Digestive Health, UCL Division of Medicine, Royal Free Campus, University College London, London, United Kingdom; Semmelweis University, HUNGARY

## Abstract

**Background:**

Classification of medical sciences into its sub-branches is crucial for optimum administration of healthcare and specialty training. Due to the rapid and continuous evolution of medical sciences, development of unbiased tools for monitoring the evolution of medical disciplines is required.

**Methodology/Principal Findings:**

Network analysis was used to explore how the medical sciences have evolved between 1980 and 2015 based on the shared words contained in more than 9 million PubMed abstracts. The k-clique percolation method was used to extract local research communities within the network. Analysis of the shared vocabulary in research papers reflects the trends of collaboration and splintering among different disciplines in medicine. Our model identifies distinct communities within each discipline that preferentially collaborate with other communities within other domains of specialty, and overturns some common perceptions.

**Conclusions/Significance:**

Our analysis provides a tool to assess growth, merging, splitting and contraction of research communities and can thereby serve as a guide to inform policymakers about funding and training in healthcare.

## Introduction

The medical sciences, like any other branch of science, exhibit a complex emergent behavior that continuously shapes the structure of medicine. New concepts inspire new subject areas, which eventually merge with other disciplines to create a bigger branch, or splinter into sub-communities to form new smaller branches. A good historical example is the rise of biochemistry, which spawned from the merger between biology and organic chemistry in the early 1900s. Biochemistry as a discipline further evolved into a variety of more specialized communities such chemical pathology, enzymology and metabolism. The selection pressure for change can be social as well as scientific: one of these new offshoots, molecular biology, resulted from a fusion of post-WWII-era physics with the more biological end of biochemistry, fueled by disillusioned physicists fleeing their field and seeking more positive subject matter in the aftermath of the Manhattan Project [1].

Although understanding the emergent behavior of the medical science is interesting within the context of the evolution of complex systems, it is also crucial for its optimum administration. Efficient medical education, budget allocation and healthcare management are strongly influenced by how medical science is formally classified into its sub-branches. If policymakers are working with out-of-date information, they will be unable to allocate their research budgets to maximum effect, or to devise effective training and educational strategies. Therefore, pragmatic, responsive tools for reliably tracking the evolution of medical disciplines are crucial.

Medical research papers are a valuable source of information that can reflect the trends of collaboration and overlap between different disciplines. The symbolic structure of research papers, especially the agreed formal vocabulary that their authors tend to use, has the potential to serve as a reliable source of information for classification of research into its sub-categories. Unlike the traditional arbitrary classification of medical lexicons, recent advances in network analysis enable us to dissect the dynamics of mutual relationships between medical words at different time intervals. Such analyses can generate a dynamic network which consists of meaningful mutual connections between symbols (medical words) and their surrounding communities.

To investigate the evolution of medical research and the formation of communities within this network, we used a text-mining approach to study ~9,000,000 abstracts that appeared in PubMed from 1980 to 2015; from this, we created a network of shared medical lexicons. The resulting entity, which we call the Medical Words Network, consists of nodes that represent medical worlds (e.g. ‘liver’, ‘cholesterol’, ‘platelet’, ‘cortisol’) and weighted links (edges) that correspond to the mutual appearance of two words in the same abstract. To calculate the weight of edges, we used the concept of “pointwise mutual information”, which indicates how much the appearance of one word tells us about another word in a given abstract [2]. The mutual information between A and B is zero if they are independent. This means that the frequency of A among abstracts also containing B is the same as the overall frequency of A. At the other extreme, if word A and B always appear together in abstracts, then all information conveyed by A is shared with B. Therefore the mutual information is the same as the information contained in word A or B alone. There is, of course, a quantifiable spectrum of shared information between these two extremes. One advantage of the mutual information approach is that it depends on the frequency and joint probability of the words rather than on the absolute number of appearances–which may vary dramatically at different timepoints as topics go in and out of fashion.

## Results and Discussion

**Fig 1:**
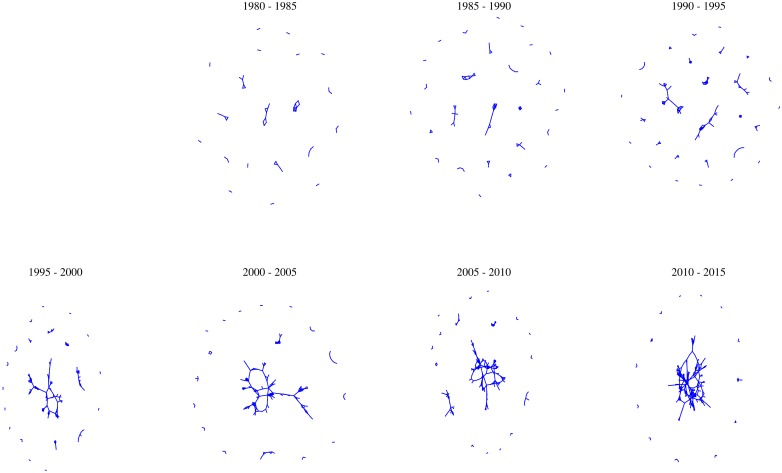
The evolution of the Medical Words Network in 1980–2015. Each node represents a medical term that has been used more than 2000 time in PubMed in each time-interval and the links indicate significant pointwise mutual information between two separate words. To consider the most important edges, a threshold for the mutual information was selected. The measuring unit of information is the bit and in this figure the pointwise mutual information more than 4 bits indicate a link between two nodes.

Fig 1 represents the evolution of the Medical Words Network between 1980 and 2015 inclusive in terms of connectivity through time. We used the graph embedding (GEM) algorithm for network visualization [3] and set two thresholds to reveal the most important nodes/links in this network (see S1 Fig for a version with the nodes labeled). Initially, in the 1980s, the network is comprised of segregated islands of small communities. Through time, these islands tend to integrate and form a more complex network. The number of satellite islands appears smaller as the central network becomes more connected and sophisticated (S2 Fig). Such evolution may reflect the influence of medical specialism in the last half of 20^th^ century, which was followed by the emergence of interdisciplinary research in recent decades [4,5]. The importance of such an interdisciplinary approach was predicted by scholars such as Wilson in the last decades of the 20^th^ century when he stated that only fluency across the boundaries will provide a clear view of the world as it really is [4].

As shown in Fig 1, from year 2000 onwards, a complex inter-connected core emerges in the heart of the network which can be the subject of detailed analysis. To analyze this core further, we employed the k-clique percolation method to identify local communities within the network [6]. Originally developed by Derényi et al., this method provides a tool to extract the local organization from within a complex network. It has been used to analyze of a variety of networks (e.g. co-authorship, phone calls and protein-protein interactions) and is based on finding communities within a network [6–8]. The advantage of this method is that the extracted communities can share nodes (as expected in real communities within a complex network)[6]. This method views a community as the union of overlapping cliques (within a graph, a clique of size k is called a k-clique). Two k-cliques are considered adjacent if they share k-1 nodes and a k-clique community is the largest connected subgraph obtained by the union of all adjacent cliques [7]. To find communities within a network a critical value for k can be optimized to avoid having a giant component that would merge many small communities and smear out the details of the community structure [7]. Guided be analogy with percolation phenomena, a giant component appears when k is below a critical point. Thus, we selected the smallest value of k for which no giant community appears as described by Palla et al. [7].

**Fig 2:**
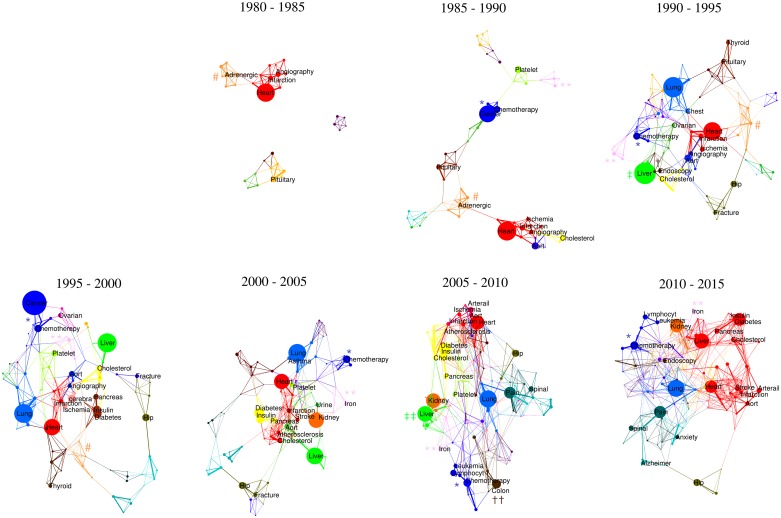
Identification of communities within the Medical Words Network based on k-clique percolation method. Each extracted community is shown with a distinct color. The k-value was set as 4 (k = 4) in order to identify the communities. The diameter of each node and the thickness of the links are proportional to the number of abstracts (for each node) and the weight (mutual information) of the link respectively.

In the next step of the study we focused on the core of the Medical Words Network and extracted its modular structure using an optimized k value. Fig 2 demonstrates evolution of the core network from 1980–2015 in terms of the communities that form. In this figure, the diameter of each node is directly proportional to the number of abstracts for each word. The frequencies of the words that were used more than 2000 times in each time interval is presented in S1 Table. The thickness of the links in Fig 2 is proportional to the weight (pointwise mutual information) of the links.

After using k-clique extraction, a variety of clusters (communities) are visible in the network which are differentiated by color (see S3 Fig for a higher resolution version of the figure). These communities share nodes and are located in close proximity to their most relevant neighbors. Between 1980 and 2015 the communities show great plasticity, and collectively exhibit the most common events that can occur in the lifetime a community (namely growth, contraction, merging, splitting, appearance and disappearance) [8]. For example, from 1990 up to 2010, two distinct clusters (metabolic [yellow] and cardiac [red]) share/exchange nodes at different time intervals. These communities eventually merge to form a bigger community in 2010–2015 (shown in red). What’s more, this new large community (2010–2015) also includes the liver (hepatology) network. From 1990–2010, the hepatology (green) and cardiac (red) clusters had minimal shared links but, during last five years, these two communities began publishing papers that significantly shared information about both disciplines. This is not surprising given the high incidence of metabolic disease in the last decade. It seems, therefore, that a public health imperative has brought together two separate communities because of their need to deal with related co-morbidities (fatty liver disease and cardiovascular disease).

As another example, hepatology is traditionally regarded as a sub-branch of gastroenterology. Indeed, according to our model, in 1990–1995, hepatology (green) and luminal gastroenterology (brown) manifest as two separate but close communities with some shared nodes (‡ and † in Fig 2). Strikingly however, in more recent years they have lost almost all shared nodes so that, by 2005–2015, the two disciplines are no longer even in the same neighborhood (‡‡ and †† in Fig 2). Despite this clear separation of communities, the two disciplines are often still lumped together; for example, in the Population and Systems Medicine Board of the UK Medical Research Council, they are regarded as one entity called ‘gastroenterology’. A related phenomenon can be observed with the hematology (pink, ** in Fig 2) and oncology (indigo, * in Fig 2) clusters. While practitioners in many countries consider these two disciplines as one specialty, the Medical Words Network almost always distinguishes these two communities as two separate clusters that overlap with only a few nodes (e.g. anaemia).

Our model indicates that, despite specialty training in most fields of medicine still being based on anatomy and physiology of organs, distinct communities within each specialty extensively collaborate with other communities within other domains of specialty, sometimes preferentially. Our analysis suggests that some medical specialty training fields today may not reflect the real research communities underpinning them, and by extension, may not provide the most efficient system to challenge current health care problems. The emphasis of modern medicine on specialism may lead to astounding developments, but equally could result in a confined point of view. Today's multidisciplinary teams working on a variety of diseases demonstrate a practical, retrospective way to solve the drawbacks of the traditional categorization of medicine–but broadening the emphasis and introducing interdisciplinarity early on in medical education could reap bigger rewards.

In addition to providing a new classification of evolving medical disciplines, network analysis can also shed light on novel concepts within medical networks at the micro, meso and macro scales. At the microscopic end, the concept of a word is altered as its local network evolves. New disciplines with a different vision can modify even the practical meaning of a word (i.e. a disease or a drug), which in turn can result in emerging novel concepts. For example aspirin, which was originally discovered as an anti-inflammatory drug, eventually became a key player in the hemostasis community (light green community in Fig 2) due to its anti-platelet effect. From 2005 till 2015, aspirin has been shared within the hemostasis, cardiovascular and metabolic communities, most likely due to its protective effect against heart attack. In turn, emerging evidence that aspirin may prevent colon cancer [9] could facilitate the merger of communities in future that seem apart today.

At the mesoscopic view, geographic borders of different fields appear to have plasticity with respect to the growth of connectivity, and this plasticity is followed by emergence and annihilation of some fields. Apart from merging and splitting which was discussed above, a community may contract and eventually disappear from the Medical Words Network. A clear example is the evolution of Autonomic Nervous System community (# in Fig 2) from 1980 until 2015. Although this community was one of the main building blocks of the Network in 1980–1985, it contracted and eventually disappeared from the Network by 2000. In addition, a new community may emerge with potential to solve a healthcare problem: Although some relatively new fields such as emergency medicine or geriatrics are known entities, there are still some fields that are practiced in the real world, but do not have an official branch–or a recognized name. This matters, because it is difficult to follow, foster and fund a field that has no name: names confer legitimacy and status. Finally, in the macroscopic view, aggregation of connections among different field could be inferred as a signal of the end of organ-based specialism. Future medicine might be better practiced in multidisciplinary clinics that are run by practitioners with a more panoramic view and training.

## Materials and Methods

Text-mining approach has been used previously for construction of networks [10–13]. In the present study network analysis was used to explore how the medical sciences have evolved between 1980 and 2015 based on the shared words contained in more than 9 million PubMed abstracts.

*Source of Data*: All abstracts in PubMed until January 2015 inclusive were uploaded using contact with Application Programming Interface (API) of the National Library of Medicine (http://www.nlm.nih.gov/api/). We only included entities with English abstracts for the analysis (9,592,193 in total) which consisted of 522195, 779545, 1028478, 1163947, 1407614, 1827149 and 2429399 items for 1980–1985, 1985–1990, 1990–1995, 1995–2000, 2000–2005, 2005–2010 and 210–2015 time-intervals respectively.

*Development of the Medical Words Network*: The software was developed in MATLAB for analysis of the network (see S1 File for codes in MATLB). Words that were used more than 2000 times were considered for further analysis and the pointwise mutual information between all permutations of two words were calculated based on the following formula [2]:

$$Pointwise\;mutual\;information\;\left(A,B\right)=log_{2}\frac{p\left(A,B\right)}{p\left(A\right).p\left(B\right)}=log_{2}\frac{\frac{f\left(A,B\right)}{N}}{\frac{f\left(A\right)}{N}.\frac{f\left(B\right)}{N}}$$

Where *p*(*A*, *B*) is the joint probability of the co-occurrence of both words A and B in the abstracts, and *p*(*A*) is the probability of observing word A among the abstracts. *p*(*A*, *B*) is calculated by dividing the frequency of abstracts containing both A and B (*f*(*A*, *B*)) by total number of abstracts (*N*) in a given time interval. Likewise, *p*(*A*) is calculated by dividing the frequency abstracts containing A (*f*(*A*)) by *N*.

To consider the most important nodes, a threshold was selected for each node (words used more than 2000 times in each time-interval). Likewise a threshold was selected for the edges. Changing the threshold is like changing the resolution with which the network structure is investigated. In this study pointwise mutual information more than 4 and 2.8 bits were selected for Figs 1 and 2 respectively. The graph embedding (GEM) algorithm was embedded for network visualization [3]. This algorithm minimizes edge crossings, allows uniform edge lengths and doesn’t allow nodes to overlap with edges that are not incident on them. The pointwise mutual information between all permutations of two words in each time interval is shared at https://dx.doi.org/10.6084/m9.figshare.4233722.

*Community detection within a network*: A variety of different methods have been developed for detection of communities within a network [14]. In this study we employed the k-clique percolation method to extract local clusters within a network. As expected in natural networks, we used a method that allows share nodes among the communities [6,7,8,14]. In brief, k-clique is a set of k nodes which all are connected directly. Two k-cliques are neighbor if they share k-1 nodes. A set of k-cliques that are all connected through at least one neighboring path comprise a community. Optimized k for extraction of local network communities was selected by calculating the percolation threshold as described [6,7]. Open source software for finding and visualizing overlapping communities in networks, based on the k-clique percolation method can be found at http://www.cfinder.org/.

## Supporting Information

S1 Fig The evolution of the Medical Words Network in 1980–2015. Each node represents a medical term that has been used more than 2000 time in PubMed in each time-interval and the links indicate significant mutual information between two separate words.
(PNG)

S2 Fig The connectivity of the Medical Words Network increases significantly through time from 1980 to 2015. (A) Size of the giant component, (B) Average distance in the largest component, (C) Number of connected components.
(PNG)

S3 Fig Identification of communities within the Medical Words Network based on k-clique percolation method (k = 4). Each extracted community is shown with a distinct color. The diameter of each node and the thickness of the links are proportionate to the number of abstracts (for each node) and the weight (mutual information) of the link respectively.
(PNG)

S1 File The software developed in MATLAB for calculation of pointwise mutual information and construction of the Medical Words Network. Please rename the file to PMINetwork.m before using is as a function. PMINetwork is a function that calculates the pairwise mutual information between two words based on their co-occurrence in the abstracts.
(M) Click here for additional data file.

S1 Table The frequencies of the words that were used more than 2000 times in 1980–1985, 1985–1990, 1990–1995, 1995–2000, 2000–2005, 2005–2010 and 210–2015 time-intervals. (XLSX)
