# Supplementary figures and images for: Evolution of Communities in the Medical Sciences: Evidence from the Medical Words Network
Source: PLoS One. 2016 Dec 2;11(12):e0167546. doi: 10.1371/journal.pone.0167546 (PMC5135137; doi:10.1371/journal.pone.0167546)

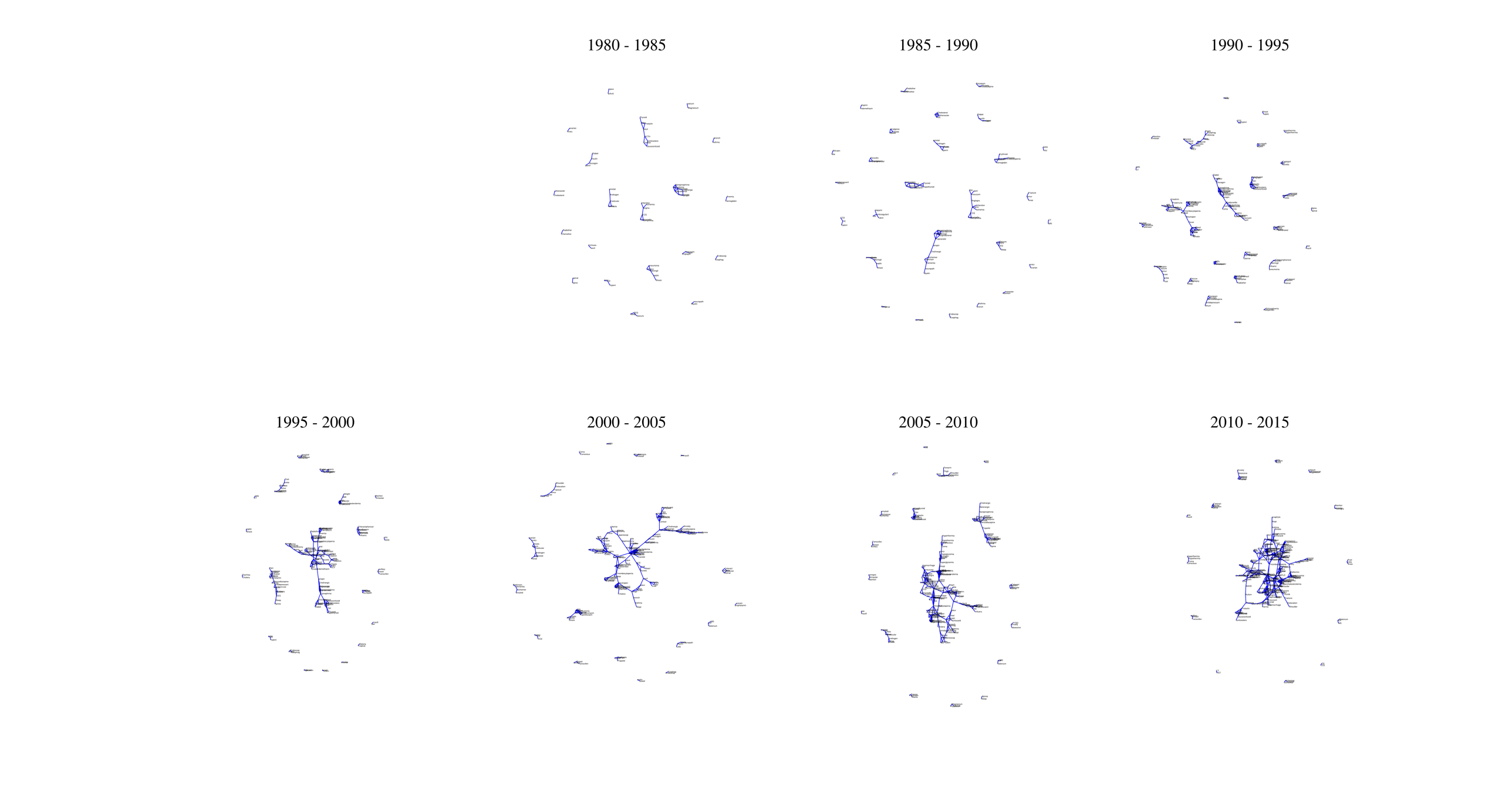

Supplement: S1 Fig — Each node represents a medical term that has been used more than 2000 time in PubMed in each time-interval and the links indicate significant mutual information between two separate words. (PNG) [file pone.0167546.s001.png]

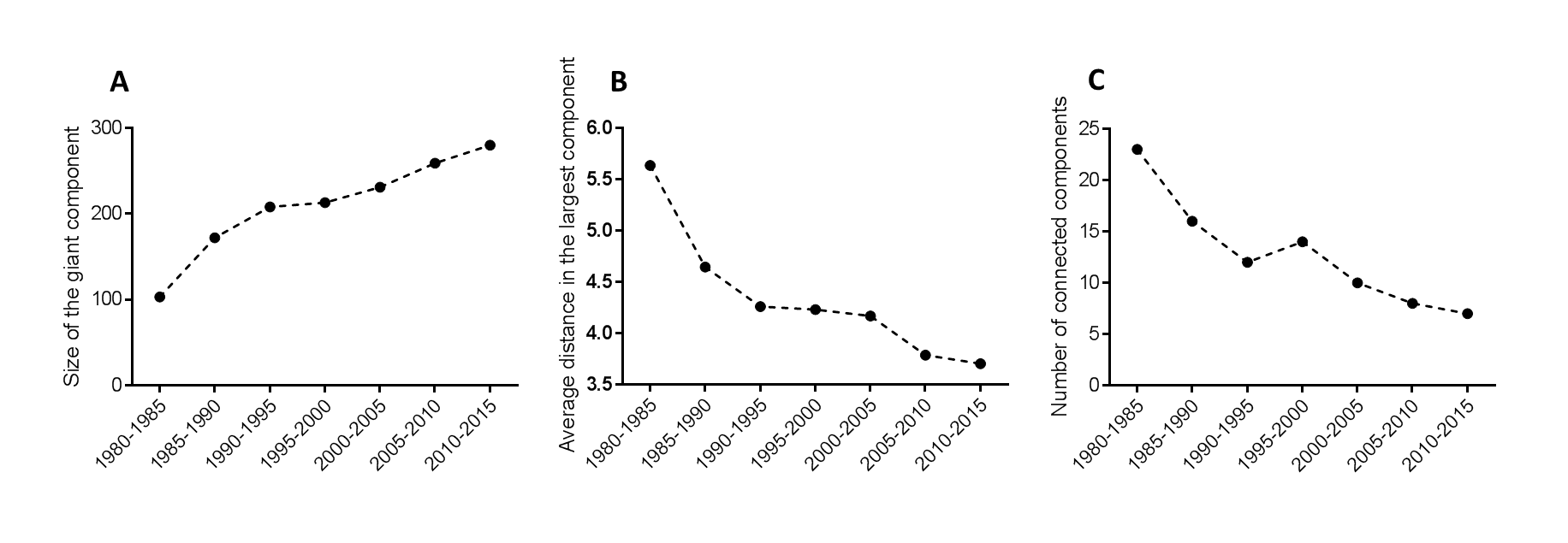

Supplement: S2 Fig — (A) Size of the giant component, (B) Average distance in the largest component, (C) Number of connected components. (PNG) [file pone.0167546.s002.png]

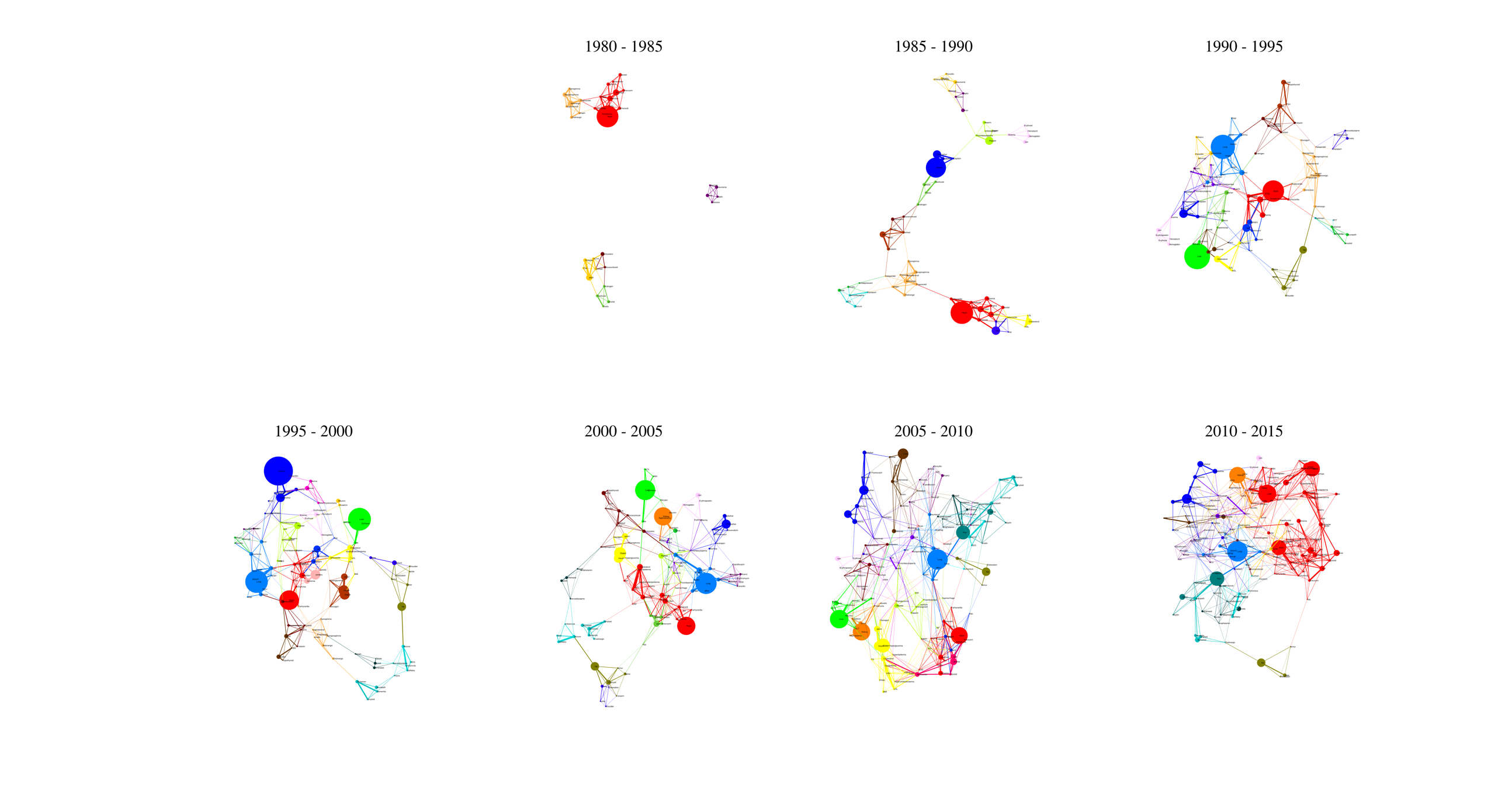

Supplement: S3 Fig — Each extracted community is shown with a distinct color. The diameter of each node and the thickness of the links are proportionate to the number of abstracts (for each node) and the weight (mutual information) of the link respectively. (PNG) [file pone.0167546.s003.png]
